# Analysis of apical hook formation in Alaska pea with a 3-D clinostat and agravitropic mutant *ageotropum*

**DOI:** 10.3389/fpls.2014.00137

**Published:** 2014-04-08

**Authors:** Kensuke Miyamoto, Takahiro Yamasaki, Eiji Uheda, Junichi Ueda

**Affiliations:** ^1^Faculty of Liberal Arts and Sciences, Osaka Prefecture UniversitySakai, Osaka, Japan; ^2^Faculty of Science, Osaka Prefecture UniversitySakai, Osaka, Japan; ^3^Graduate School of Science, Osaka Prefecture UniversitySakai, Osaka, Japan

**Keywords:** *ageotropum*, apical hook, auxin polar transport, clinostat, epicotyl bending, microgravity, *Pisum sativum*

## Abstract

The formation of the apical hook in dicotyledonous seedlings is believed to be effected by gravity in the dark. However, this notion is mostly based on experiments with the hook formed on the hypocotyl, and no detailed studies are available with the developmental manners of the hook, particularly of the epicotyl hook. The present study aims at clarifying the dynamics of hook formation including the possible involvement of gravity. Time-course studies with normal Alaska pea (*Pisum sativum* L., cv. Alaska) and an agravitropic pea mutant, *ageotropum*, under the 1-*g* conditions and on a 3-D clinostat revealed that (1) the apical hook of the epicotyl forms by the development of the arc-shaped plumule of the embryo existing in the non-germinated seed. The process of formation consists of two stages: development and partial opening, which are controlled by some intrinsic property of the plumule, but not gravity. Approximately when the epicotyl emerges from the seed coat, the hook is established in both pea varieties. In Alaska the established hook is sustained or enhanced by gravity, resulting in a delay of hook opening compared with on a clinostat, which might give an incorrect idea that gravity causes hook formation. (2) During the hook development and opening processes the original plumular arc holds its orientation unchanged to be an established hook, which, therefore, is at the same side of the epicotyl axis as the cotyledons. This is true for both Alaska and *ageotropum* under 1-*g* conditions as well as on the clinostat, supporting finding (1). (3) Application of auxin polar transport inhibitors, hydroxyfluorenecarboxylic acid, naphthylphthalamic acid, and triiodobenzoic acid, suppressed the curvature of hook by equal extents in Alaska as well as *ageotropum*, suggesting that the hook development involves auxin polar transport probably asymmetrically distributed across the plumular axis by some intrinsic property of the plumule not directly related with gravity action.

## INTRODUCTION

The apical hook is the arc-shaped transient structure formed in seed germination process on top of the hypocotyl or epicotyl of dicotyledonous seedlings. It is believed that, when seeds germinate in the field, the apical hook is formed in the dark in soil and opens in response to light near the surface of soil, thus plays a role to protect the fragile apical meristem from possible injuries when passing through the soil (Taiz and Zeiger, 2010). When the hook is formed in the dark, that gravity plays a key role was shown in sunflower, cress and cucumber (MacDonald et al., 1983; Takahashi and Suge, 1988) by means of a clinostat or other means.

The advent of experiments in a spacecraft or a space station made it possible to compare the growth and development of plants under 1-*g* conditions on the earth with those under the microgravity ones in space to learn the effects of gravity (see Halstead and Dutcher, 1987; Hoson and Soga, 2003; Paul et al., 2013). In the STS-95 space experiments, NASA, the present authors also joined, and discovered that Alaska pea seedlings grown in the dark in space developed the epicotyl in an oblique upward direction away from the cotyledons and elected the root also in an upward direction asymmetric to the epicotyl. Besides the peculiar morphology of the shoot and root, the apical hook was also found to be markedly reduced in curvature (Ueda et al., 1999, 2000). A similar abnormal growth pattern of a seedling was observed to occur in an agravitropic pea mutant, *ageotropum*, under 1-*g* conditions in the dark (Schöldéen and Burström, 1960; Olsen and Iversen, 1980a,b; Strudwick et al., 1997). The anomalous shape occurs not at random but uniformly in the majority of seedlings tested, leading to the idea that it is regulated by some intrinsic property of the seedlings, which is manifested first when the action of gravity is removed. This concept was already proposed by Pfeffer (1904) as automorphosis (Eigenrichtung; reviewed by Stanković et al., 1998) and served for explaining the establishment of intracellular polarity and determination of the growth direction in space (Volkmann et al., 1986) or on a clinostat (Hoson et al., 1992, 1996, 1997).

As stated in the first paragraph, most experiments on the apical hook formation used epigeal plants as materials, the hypocotyl of which raises the hook upto near the soil surface; while in hypogeal plants no detailed studies on the apical hook formation are available. Furthermore, most studies were concentrated on the hook already established on an elongated hypocotyl, but rarely dealt with the process of hook development. The findings that Alaska pea seedlings formed the apical hook in space or on a clinostat, even if less developed, suggest that development of the apical hook may be caused by some intrinsic property (automorphosis) of pea seedlings besides gravity. In such background the present study aims to clarify how the apical hook develops, and how the intrinsic property and/or gravity are involved in the hook development. To achieve the aims the whole process of hook development is followed under the 1-*g* conditions in comparison with that obtained on a 3-D clinostat. The same experiments are carried out with *ageotropum*, to provide another control in addition to the one on a clinostat. Finally, the possible involvement of auxin polar transport is examined with relevant inhibitors. The above-planned analyses of the apical hook of pea seedlings are to increase understanding of apical hook formation in the hypogeal seedlings which has seldom been investigated.

## MATERIALS AND METHODS

### PLANT MATERIALS AND CULTURE

Two kinds of pea plants, *Pisum sativum* L., cv. Alaska and an agravitropic mutant, *ageotropum*, were used. Seeds of Alaska were purchased from Watanabe Seeds, Misato, Miyagi, Japan and seeds of *ageotropum* were propagated in the experimental field of the laboratory from the seeds kindly supplied by Prof. Hideyuki Takahashi, Tohoku University, Sendai, Japan. As seed bed, rock wool blocks, 9 cm × 4.8 cm × 1.5 cm, cut out from a large sheet of rock wool (Chibikko Ace Mat, Nippon Rockwool Co. Ltd., Tokyo, Japan) were individually placed in acrylic resin boxes (9 cm × 4.8 × cm × 5.8 cm) of an exactly fitting size. For ventilation each box had four holes, 1 cm in diameter, in the ceiling, and the holes were covered with hydrophobic fluoropore membrane (MilliSeal, Millipore, Merck). On the seed beds so prepared, 12 seeds each were set in the manner that a whole seed was buried beneath the block surface, and the seed axis (the line to connect the plumular axis and radicle) was normal to the upper surface of the block. After supplied with 40 ml water, each box was placed in a zipper-locked bag and kept at 23.5°C in the dark under 1-*g* conditions or on a three-dimensional clinostat (3-D clinostat).

### 3-D CLINOSTAT

It was manufactured by Nihon Ikakikai, Ltd., Osaka, Japan according to the original design by Hoson et al. (1988, 1992) and its operation was controlled with a rotation control system (Model CL-CS1, Minamide System Engineering, Ltd., Osaka, Japan). The clinostat system was composed of a clinostat within which another clinostat was equipped, and both clinostats were rotated independently at a variable rate up to 2 rpm, changing the rate and direction of rotation so that the gravity action integrated in all directions was null.

### DETERMINATION OF APICAL HOOK AND EPICOTYL BENDING

**FIGURE 1:**
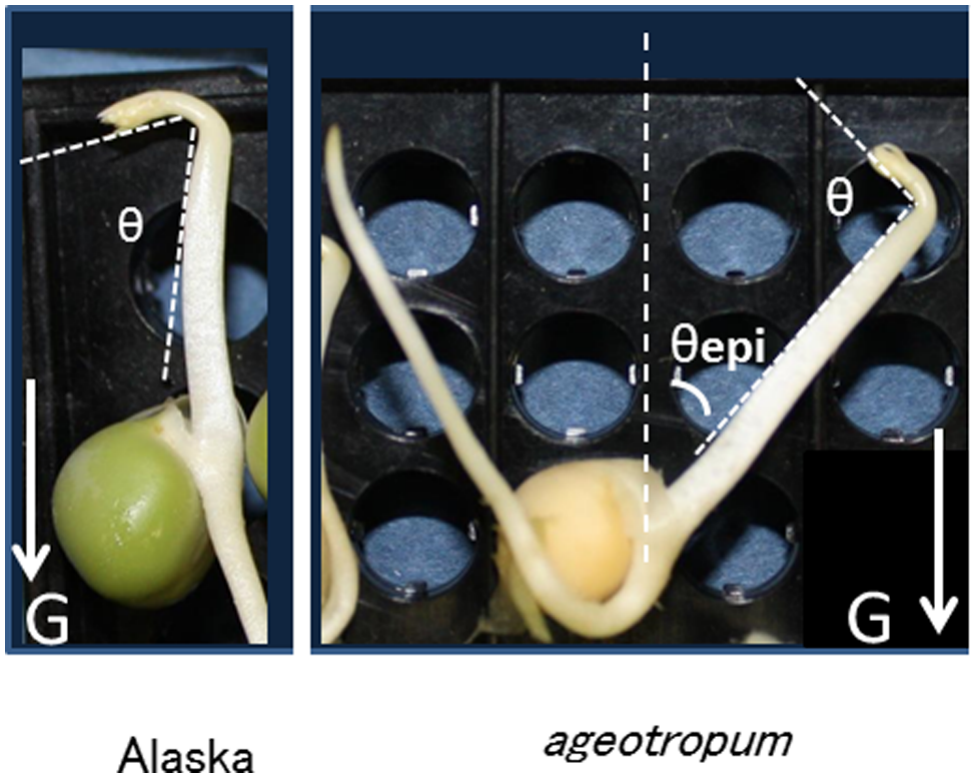
**Pea seedlings, cv. Alaska and agravitropic mutant, *ageotropum*, grown under 1-*g* in the dark at 23.5°C for 84 h asw (after supplying water to dry seeds), and the definition of hook angle (θ) and epicotyl bending (θ_epi_).** The G arrows indicate the direction of gravity. In *ageotropum* the epicotyl extends in the direction of about 40° away from the seed axis, and the root elongates in the symmetric direction to the epicotyl. Note that the apical hook bends on the same side of the epicotyl as the cotyledons in both pea varieties.

Seedlings grown as above were harvested and photographed at the time(s) indicated, and the angles of apical hook and epicotyl bending were determined with a protractor on enlarged photographs. As shown in **Figure 1**, the apical hook angle represents the angle formed by the straight parts above the apical hook and the sub-apical epicotyl part mainly consisting of the elongation zone, and the epicotyl bending, the angle between the seed axis and the lower straight part of the epicotyl. To find the seed axis easily, a needle was stood in the gap of the cotyledons prior to being photographed (**Figure 7**).

### LOCALIZATION OF AMYLOPLASTS

Seedlings standing on a growth bed served for staining. On one side of the epicotyl a longitudinal incision spanning from the lower end of the apical hook to about 10 mm below was made, and a drop of I_2_–KI solution was applied to the incision and allowed to diffuse into tissues. After 5 min when the tissues were fixed with the staining reagent, the epicotyl was sliced into about 100 μm thick pieces with a razor blade by hand and photographed under a light-microscope (Olympus BH2, Tokyo). The method was according to Scott (1988).

### APPLICATION OF INHIBITORS

The tested inhibitors were auxin polar transport inhibitors, 9-hydroxyfluorene-9-carboxylic acid (HFCA), *N*-(1-naphthyl) phthalamic acid (NPA), and 2,3,5-triiodobenzoic acid (TIBA). They were purchased from Sigma (St. Louis, MO, USA) or Tokyo Kasei Kogyo Ltd. (Tokyo, Japan) and used without further purification. They were individually dissolved in water at 10 μM, and applied, instead of plain water for starting germination, to dry rock wool where dry seeds had already been buried.

## RESULTS

### AGRAVITROPIC MUTANT *AGEOTROPUM* MIMICS ALASKA PEA SEEDLINGS GROWN UNDER MICROGRAVITY CONDITIONS

**FIGURE 2:**
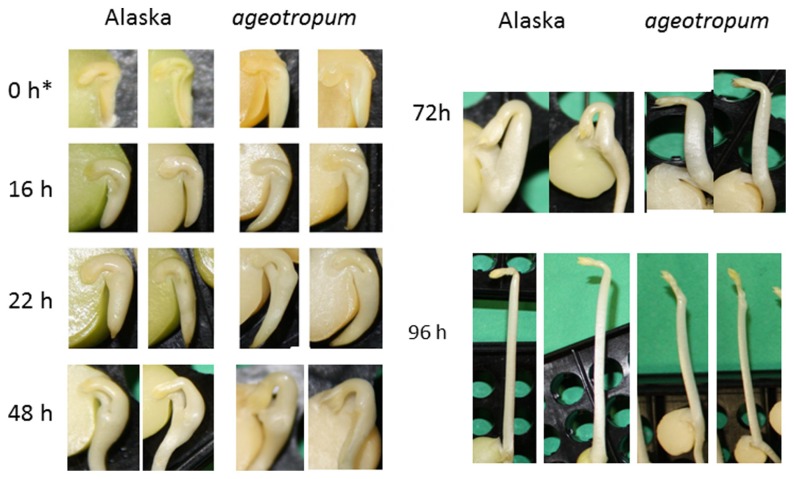
**Kinetics of the apical hook development in Alaska and *ageotropum* pea seedlings grown under 1-*g* conditions.** Photographs were taken at the time points shown in h asw. To show the inside, the cotyledon at the front side was removed prior to photographing. Photos at 0 h* show seeds imbibed in water in a refrigerator for 1 h to facilitate dissection. Although the epicotyl of *ageotropum* extended obliquely under 1-*g* conditions as well, the photos at 72 h and 96 h asw are arranged so that the epicotyl parallels that of Alaska for the convenience of comparing the hooks between the two varieties. Time is in h asw.

Under the microgravity in space and a simulated microgravity on the 3-D clinostat in the dark, etiolated pea seedlings of normal cultivar Alaska represented abnormal growth and morphology, i.e., the epicotyl bearing the partially opened apical hook grew in the oblique direction deviated by about 40° away from the cotyledons and the root elongated in the oblique upward direction symmetric to the epicotyl. In order to learn how seedlings of the agravitropic pea mutant *ageotropum* grown under 1-*g* conditions simulates such abnormal growth and morphology that Alaska seedlings showed in space or on a clinostat, *ageotropum* seedlings were grown in the dark under 1-*g* conditions and their growth and morphology were followed in comparison with Alaska seedlings during the germinating process for 96 h asw (after supplying water to dry seeds; **Figures 1** and **2**).

**FIGURE 3:**
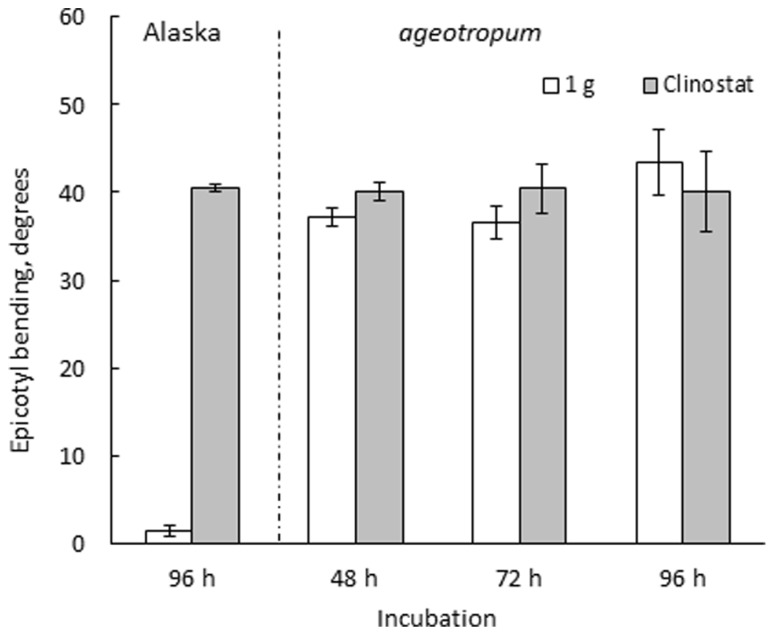
**Effects of 3-D clinostat rotation on the epicotyl bending in Alaska and *ageotropum* seedlings.** Data bars: means with standard errors (*n* = 10); time: h asw.

Seedlings of both varieties were harvested at intervals and photographed to collect data. *Ageotropum* seedlings showed abnormal orientation of the root and shoot, and reduced hook development, all of which were similar to those of Alaska observed under the microgravity conditions in space (Ueda et al., 1999, 2000) and on a 3-D clinostat (Miyamoto et al., 2005a,b, 2007). The epicotyl bending in *ageotropum* under 1-*g* conditions appeared already at 48 h asw when the epicotyl began to elongate and was maintained at least until 96 h asw (**Figure 3**). Furthermore it was not affected by rotation on a 3-D clinostat. Thus, *ageotropum* seedlings were confirmed to be non-responsive to gravity and mimic Alaska seedlings grown under microgravity conditions, providing a criterion for inferring the gravity-related responses in apical hook development.

**FIGURE 4:**
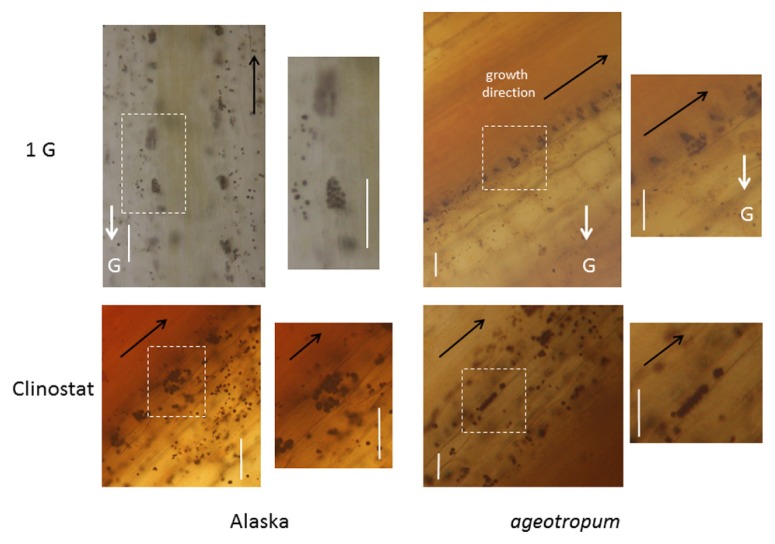
**Intra-cellular localization of amyloplasts in cv. Alaska and *ageotropum* under 1-*g* conditions and on a 3-D clinostat.** The photographs are of sections prepared from the epicotyl part about 2 mm below the lower end of the apical hook. Dark violet stains are amyloplasts, which have sedimented along the direction of gravity, G-arrows, under the standstill 1-*g* conditions in cv. Alaska as well as *ageotropum*, but not on the clinostat; note that in *ageotropum*, gravity worked obliquely to the epicotyl since it slanted. Scale bars: 100 μm.

Fresh, iodine-stained longitudinal sections revealed that *ageotropum* seedlings appeared to have a normal content of amyloplasts which sedimented in the direction of gravity as observed in Alaska seedlings (**Figure 4**). The result is similar to that reported with the root by Olsen and Iversen (1980b). The microscopic observation suggests that the amyloplasts and their sedimentation in epicotyl of *ageotropum* are normal, but its gravity perception/transduction system is considered to be disturbed in a step(s) other than amyloplast sedimentation.

### DEVELOPMENT OF APICAL HOOK IN ALASKA AND *AGEOTROPUM* PEAS UNDER 1-*g* CONDITIONS

**FIGURE 5:**
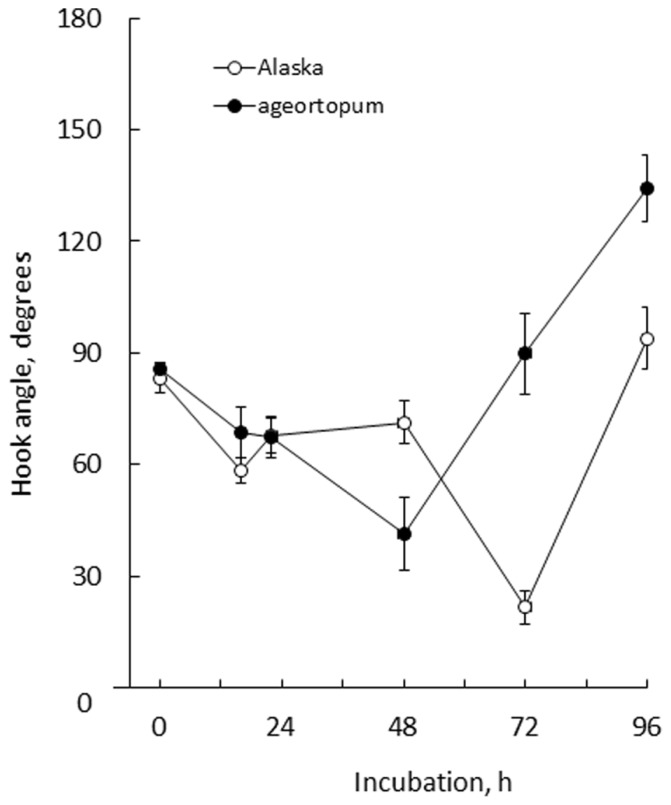
**Kinetics of the development of apical hooks in Alaska and *ageotropum* seedlings under 1-*g* conditions.** Data points: the means with standard errors (*n* = 10); time: h asw.

In Alaska pea, the arc-shaped plumule of embryo having an angle of about 90° has already been formed in the embryo in dry seeds (0 h^*^Figure 2). Kinetic observation under 1-*g* conditions revealed that the apical hook was derived from the arc of plumule. As the epicotyl grew, the arc also developed intensifying its curvature, i.e., decreasing the angle of arc, by faster growth on the distal side to the cotyledons (outer side) than on the proximal side (inner side) from 22 to 48 h asw (**Figure 2**). Accordingly, the apical hook bent on the same side of the epicotyl as the cotyledons, or geometrically expressed the apical hook and the cotyledons shared a plane containing the epicotyl axis. A maximal curvature of the hook was reached when the epicotyl was ca. 5~10 mm long (72 h asw; **Figures 2** and **5**). Then, as the epicotyl elongated further, hook angle increased, i.e., hook opened partially even in the dark under 1-*g* conditions.

**FIGURE 6:**
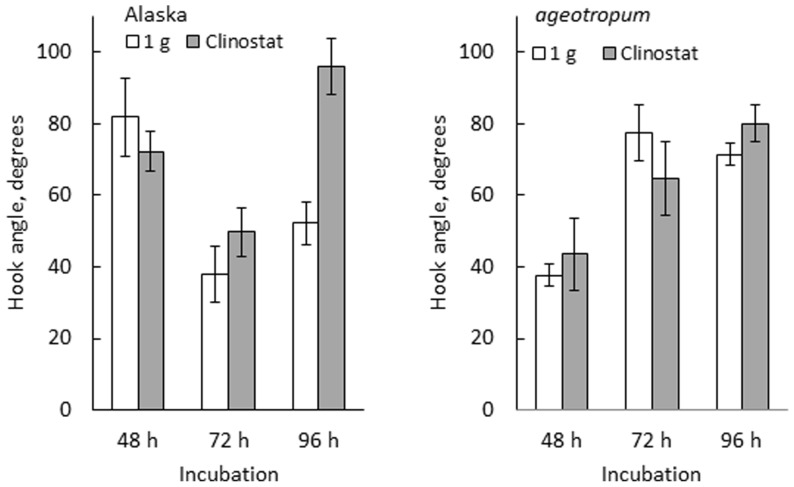
**Effects of clinostat rotation on the hook development in Alaska and *ageotropum*.** Data bars: the means with standard errors (*n* = 8–10); time: h asw.

In *ageotropum*, an arc-shaped structure of plumule of the embryo in dry seeds gave rise to the apical hook similarly to the case in Alaska up to 48 h asw (**Figures 2**, **5**, and **6**). Subsequently, however, the hook shifted into the opening phase without such sustention or enhancement of hook as observed in Alaska. The sustention or enhancement of the hook found from 48 to 72 h asw is characteristic to the hook development in Alaska. **Figure 2** shows, however, that Alaska seedlings developed the hook slightly slower than *ageotropum*. Hence one might think that the delayed hook development of Alaska may have reflected to the difference in hook angle between Alaska and *ageotropum* 48–72 h asw, but this possibility will be removed by the subsequent experiments.

### APICAL HOOK DEVELOPMENT ON A 3-D CLINOSTAT

The developmental path of the apical hook in Alaska was followed on the clinostat under 1-*g* conditions from 0 to 96 h asw in comparison with that observed under standstill 1-*g* conditions. In parallel, a similar experiment was also performed on *ageotropum* (**Figure 6**). In Alaska, until the hook was established at 72 h asw, no significant effect of clinostat rotation was noticed. In subsequent 24 h, however, the established hook reduced its curvature markedly on the clinostat, whereas under the standstill conditions it maintained its sharp angle of arc. In *ageotropum*, by contrast, no significant effect of the clinostat was observed, as was expected from its non-responsiveness to gravity. Thus, the sustention of the curvature of the established apical hook observed in Alaska under 1-*g* conditions from 48 to 72 h is inferred due to gravity.

### EFFECT OF AUXIN POLAR TRANSPORT INHIBITORS

**FIGURE 7:**
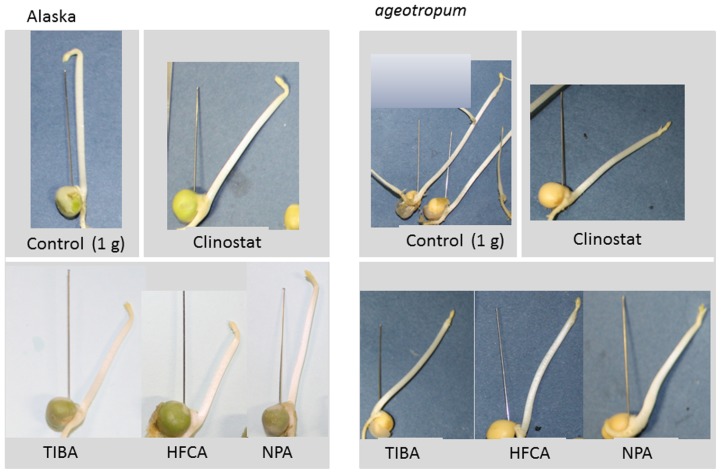
**Effects of auxin polar transport inhibitors on the hook development and the epicotyl bending in Alaska and *ageotropum*.** Aqueous solutions (10 μM) of inhibitors were individually added to the rock wool blocks embedding dry seeds, and seedlings were grown under 1-*g* conditions for 96 h asw. Plain water controls were grown under 1-*g* conditions and also on the 3-D clinostat. To indicate the seed axis the needle was set up in the gap between the cotyledons prior to photographing.

In order to see if the apical hook development involves auxin transport, three inhibitors of auxin polar transport, TIBA, NPA, and HFCA, were individually tested on seedlings of cv. Alaska and *ageotropum* under 1-*g* conditions. Each inhibitor solution at 10 μM was supplied to substrata in which seeds had been set and the results were determined after 96 h. For control, plain water was given. Fortunately, these inhibitors at the concentration used did neither affect seed germination, nor epicotyl elongation. As reported by Miyamoto et al. (2005b), the inhibitors caused epicotyl bending in cv. Alaska to the extent of 80% of plain water control on a clinostat (**Figure 7**), indicating that the inhibitor treatments were effective in inducing the epicotyl bending.

**FIGURE 8:**
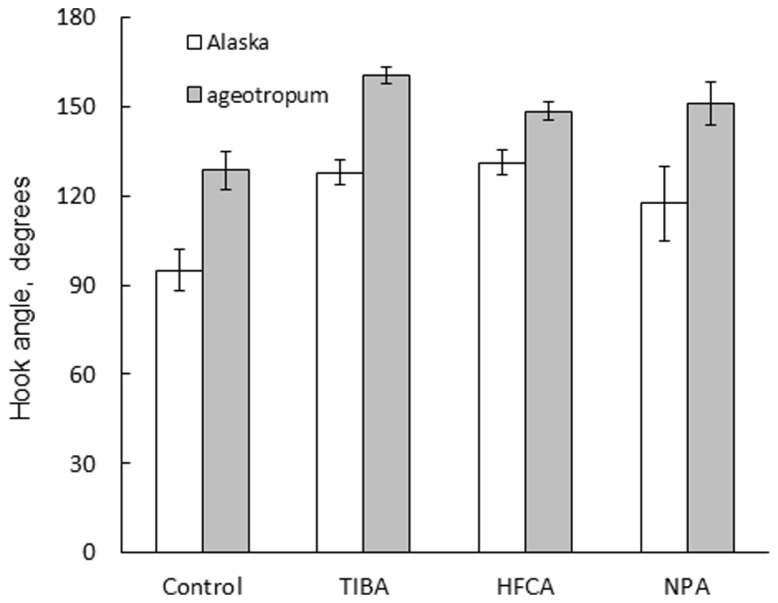
**Effects of auxin polar transport inhibitors on the hook development in Alaska and *ageotropum*.** Data bars: the means with standard errors (*n* = 10).

In addition, the same treatments reduced the extent of apical hook, i.e., opened the hook in Alaska nearly to the extent of the hook observed on the clinostat (**Figure 7**). Interestingly, the hook of *ageotropum* seedlings also was caused to open by percentages similar to those observed in Alaska (**Figure 8**). These findings indicate that auxin polar transport is involved in hook development and maintenance of the apical hook in Alaska as well as *ageotropum*. Being equally effective in both varieties of pea seedlings suggests that auxin polar transport controls hook development caused by the intrinsic property of seedlings independently of gravity, but it is not clear if the gravity-controlled phase of hook development, i.e., the enhancement and/or maintenance of the hook by gravity is also the case.

## DISCUSSION

In the present study, the role of gravity in the formation and development of the epicotyl apical hook of dark-grown pea seedlings was examined by rotation on a 3-D clinostat and comparison with the agravitropic pea mutant, *ageotropum*. The clinostat is designed and rotated at an appropriate rate so that the test plants mounted are uniformly subjected to gravity in all directions, hence integrated gravity of null (Hoson et al., 1992, 1996, 1997). Tests with the growth and development of seedlings of several species including pea have shown that the clinostat mimics the microgravity conditions in space (Kraft et al., 2000; Hoson and Soga, 2003). In fact, the shape of Alaska pea seedlings on the clinostat in the dark, the slanted epicotyl shown in **Figure 7** for example, is similar to that observed in space (Ueda et al., 1999, 2000). The shape of *ageotropum* seedlings grown under 1-*g* conditions in the dark also is similar to that of Alaska obtained in space (**Figures 1** and **6**, Ueda et al., 1999, 2000). Thus, the use of the 3-D clinostat and *ageotropum* mutant is sufficiently qualified methods to examine the role of gravity on the development of the apical hook of pea seedlings on the ground.

The apical hook of pea seedlings is formed by the development of the arc-shaped plumule of embryo in the dark, accompanied by elongation of the epicotyl (**Figure 2**). Its formation process may be divided into two stages: development and partial opening. At the former stage the arc-shaped plumule develops to establish the hook, intensifying the curvature of arc, and at the latter stage the established hook opens partially even in the dark. It is noteworthy that both formation stages of the apical hook can take place independently of gravity, therefore do not require gravity, as shown by the experiments on a clinostat as well as with *ageotropum* (**Figure 6**). Under the 1-*g* conditions the established hook of cv. Alaska is sustained or enhanced before starting to partially open, therefore delayed to open compared with ones on a clinostat or *ageotropum* (**Figures 2**, **5**, and **6**). If judged at the latter stage, 72–96 h asw when the hook is established and the epicotyl starts vigorous growth (**Figures 2** and **6**), the hook formation might be recognized to be caused by gravity, but it is not correct. Gravity only enhanced or sustained the hook developed by the intrinsic nature of the plumule. Whether gravity works for an early limited period of or throughout the opening stage is not clear from the results obtained in the present studies. In any case, at least in pea seedlings the hook formation is due to some intrinsic property of the embryo plumule, and gravity is only to sustain or enhance the established hook.

Certainly the hypocotyl of sunflower seedling placed in a horizontal position formed its apical hook in response to gravity, and cress seedlings rotated on a clinostat formed no hook (MacDonald et al., 1983). Persimmon seeds sown in various directions produced all the downward-curved hook, except for seeds placed vertically with the micropyle end down, where the hypocotyl raised the seed part straight up without forming the apical hook until the hypocotyl received a diverged gravity owing to circumnutation of the hypocotyl top (Shichijo and Hashimoto, 2013). Thus, apical hook formation in the hypocotyl of several epigeal plants is caused by gravity. In hypogeal plants, on the other hand, to determine whether the manner of hook formation found in the present studies is the characteristic of epicotyl hooks must await accumulation of data with other hypogeal plants.

The hook development of *Arabidopsis* seedlings has been differentiated into three stages: formation, maintenance, and opening (Raz and Ecker, 1999; Vandenbussche et al., 2010; Žádníková et al., 2010). However, relating the stages of peas to those of *Arabidopsis* is difficult at present. Noteworthy is that both cases have the stage where the hook opens even in the dark.

All of the findings stated above concerning the apical hook of peas lead us to assume that differential growth between the inner and outer sides of the plumular arc is controlled by some intrinsic properties probably of the plumule itself. The property-driven differential growth is not influenced by gravity until the hook is established, but is subsequently caused to sustain or enhance the hook (**Figure 5**). At the latter stage, where the hook has established and obtained responsiveness to gravity, if the seedling is turned upside down to apply gravity inversely, what will happen to the hook? This tempting question must be left to future studies.

Another example of morphogenesis by an intrinsic property in peas is the epicotyl bending which is found in cv. Alaska placed in space or on a clinostat (Ueda et al., 1999, 2000; Miyamoto et al., 2005a,b, 2007) or in *ageotropum* and described as automorphosis [Eigenrichtung by Pfeffer (1904); see the review by Stanković et al. (1998), Volkmann et al. (1986)]. The epicotyl bends about 40° when it just starts to grow. This phenomenon may be explained as follows: an asymmetric growth of the epicotyl caused by some intrinsic property of the epicotyl takes place only for a limited time when the epicotyl starts to grow at the germination stage. In Alaska under 1-*g* conditions the intrinsic property is overcome by the influence of gravity or gravity-driven auxin transport and no epicotyl bending takes place.

Development of the apical hook is caused by differential elongation between the outer and inner sides of the plumular arc in the embryo, and has been reported to involve cell division and elongation (Raz and Koornneef, 2001), being controlled by various plant growth hormones including ethylene (Bleecker et al., 1988; Bleecker and Schaller, 1996; Lehman et al., 1996; Raz and Ecker, 1999; Vriezen et al., 2004; De Grauwe et al., 2005; Vandenbussche et al., 2010; Žádníková et al., 2010; Gallego-Bartolomé et al., 2011; Willige et al., 2012). Recent studies tend to indicate that these growth regulators exert their effects at the end through asymmetric distribution of auxin (Vandenbussche et al., 2010; Žádníková et al., 2010; Abbas et al., 2013). The present study showed that the apical hook formation was suppressed by auxin polar transport inhibitors, TIBA, NPA and HFCA, to almost the same extent in cv. Alaska as in *ageotropum* under 1-*g* conditions (**Figure 8**). The results suggest that polar transport of auxin distributed asymmetrically by the intrinsic property of the embryo plumule plays a role in the portion of the apical hook development which takes place independently of gravity. If the sustention or enhancement of the established hook by gravity (**Figures 5** and **6**) also involves auxin polar transport, it may readily be explained by possible downward translocation of auxin across the plumular axis, which has a horizontal portion (cf. **Figure 2**). That an apical hook requires higher auxin concentration at the inner than the outer side is an established knowledge (Abbas et al., 2013).

In a summary, time-course studies with normal Alaska pea and the agravitropic pea mutant, *ageotropum*, under 1-*g* conditions and on the 3-D clinostat revealed that (1) the apical hook of the epicotyl forms by development of the arc-shaped plumule of the embryo existing in the non-germinated seed. The process of formation consists of two stages: development and partial opening, and controlled by some intrinsic property of the plumule. Approximately when the epicotyl emerges from the seed, the hook is established in both pea varieties. In Alaska the established hook is sustained or enhanced by gravity, resulting in a delay of hook opening compared with on a clinostat, which might give an incorrect idea that gravity causes hook formation. Application of auxin polar transport inhibitors suppressed the curvature of hook in Alaska as well as in *ageotropum*, suggesting that the formation of the hook involves auxin polar transport independently of gravity action.

## Conflict of Interest Statement

The authors declare that the research was conducted in the absence of any commercial or financial relationships that could be construed as a potential conflict of interest.
